
JOURNAL INFORMATION
==============================
NLM Title Abbreviation: ScientificWorldJournal
Journal ID: ScientificWorldJournal
NLM Journal ID: 101131163
Journal ID: TSWJ

The Scientific World Journal

ISSN: 2356-6140
EISSN: 1537-744X
Publisher: Wiley

ARTICLE INFORMATION
==============================
PMCID: PMC6009609
PMID: 29973857
DOI: 10.1100/tsw.2002.827
Article ID: 803715
Article version: 1
Subjects: Founding Editorial

Founding Editorial - Learning and Memory

LeDoux Joseph *
Collingridge Graham

*Joseph LeDoux: ledoux@cns.nyu.edu
Publication date: 2002
Electronic publication date: 2002 Jun 20
Volume: 2
First page: 1685
Last page: 1688
Received 2002 May 13; Accepted 2002 May 13
Copyright: Copyright © 2002 Joseph LeDoux and Graham Collingridge.
Copyright year: 2002
License: This is an open access article distributed under the Creative Commons Attribution License, which permits unrestricted use, distribution, and reproduction in any medium, provided the original work is properly cited.
License URL: https://creativecommons.org/licenses/by/3.0/

Keywords: short-term memory, long-term memory, hippocampus, synaptic plasticity, amygdala, long-term potentiation

==============================
**Founding Editorial**
*TheScientificWorldJOURNAL* (2002) 2, 1685–1688
ISSN 1537-744X; DOI 10.1100/tsw.2002.827

### Founding Editorial – Learning and Memory

Joseph LeDoux* and Graham Collingridge
*Principal Editors, Learning and Memory Domain*

E-mail: ledoux@cns.nyu.edu

*Received May 13, 2002; Accepted May 13, 2002; Published June 20, 2002*

**KEY WORDS:** short-term memory, long-term memory, hippocampus, synaptic plasticity, amygdala, long-term potentiation

**DOMAINS:** learning and memory

Late in life, grappling with a failing capacity to recall his past experiences, the Spanish surrealist filmmaker, Luis Bunel, exclaimed: "Life without memory is no life at all." Indeed, we are our memories — without them we are nothing. It is thus fitting that *TheScientificWorldJOURNAL* has developed a Domain devoted to the topic of learning and memory, and we are pleased to be the Principal Editors of this endeavor.

Simply defined, memory is the storage of experiences by the brain, and learning is the process by which the storage is initiated. Thus, learning occurs during the experience, and memory afterwards.

It was once thought that learning/memory was a single capacity of the brain mediated by a single brain system. Indeed, the word memory is, in the minds of many, synonymous with the brain region called the hippocampus, which was first implicated in memory in the 1950s through studies of patients who lost their ability to remember after surgical removal of this region to treat epilepsy. However, over the years, it became apparent that only some aspects of learning and memory were affected, specifically those aspects involved with conscious or explicit memory.

We now know that many different systems in the brain are able to learn during experiences and to store information about different aspects of the experience. Most of the learning and storage occurs unconsciously or implicitly. This multiplicity of memory is sometimes interpreted to mean that the brain has many memory systems. However, an alternative and more accurate view is that these implicit systems are not memory systems *per se*. That is, these systems were not designed by evolution for the purpose of storing information. They were instead designed to accomplish certain tasks necessary for survival (i.e., sensing the world, controlling movements, detecting and defending against danger, identification and response to food objects or sexual partners, etc). Learning and memory are not their jobs, but features that allow them to do their jobs more effectively.

*Corresponding author.
©2002 with author.

One of the most fundamental facts about memory, a fact that applies across the various systems that engage in memory, is that it occurs in stages. The initial stage triggered during learning is called short-term memory (STM). In this stage, memories are fleeting and unstable, and can be disrupted in various ways. But if a memory survives without significant disruption for several hours, it goes into a more stable state called long-term memory (LTM) that is insensitive to disruption and relatively stable. This process of converting labile STM to stable LTM is generally called consolidation. When we activate a previously consolidated past experience we thus retrieve information from LTM.

It has been known for some time that memory consolidation depends on the synthesis of macromolecules. Thus, in the presence of drugs that block transcription or translation, STM is intact but does not get consolidated into LTM. Forty years ago, findings such as these led to the view that memories are stored in the form of macromolecules. Today, however, it is believed that macromolecules are necessary for LTM not because they encode memories themselves but because they are necessary to stabilize changes in synaptic connectivity that are initiated during learning and that ultimately embody the memory.

Brain scientists had been proposing that changes in synaptic connectivity underlie memory since the late 19th century. Included in the list of proponents were key neuroscience figures including Sigmund Freud, Santiago Ramon y Cajal, and Sir Charles Sherrington. The most specific hypothesis came in the mid-20th century from Donald Hebb who proposed that when two neurons are connected and one takes part in the firing of the other, the synaptic connection between the two changes such that the ease with which the first fires the other is enhanced.

In the late 1960s, the first evidence that synaptic change occurs during learning was obtained in studies by Eric Kandel and his colleagues. Kandel believed that memory needed to be understood at the synaptic level, and that the only way to achieve this kind of understanding would be through the study of a relatively simple nervous system in which the circuit underlying the behavior could be identified, the synaptic changes mediating the memory in the circuit could be pinpointed, and the molecules necessary of the synaptic changes discovered. Kandel shared the 2000 Nobel Prize for his elegant studies using this approach.

About the same time Kandel began his studies of learning in an invertebrate, a discovery was made that paved the way for studying synaptic plasticity in the mammalian brain. Tim Bliss and Terje Lomo found that certain patterns of electrical stimulation of inputs to the hippocampus led to a long-lasting facilitation of synaptic transmission in this neural pathway. Because these changes occurred in the hippocampus, this seemed to be a way to explore the basis of synaptic plasticity in a part of the brain believed to necessary to an extremely important form of human memory. The fact that so-called long-term potentiation (LTP) worked in a manner remarkably similar to Hebb's hypothesis strengthened this line of thought. Additionally, the discovery that the reason this is so is because of the way a certain class of excitatory amino acid receptors, called NMDA receptors, operate has made LTP very attractive as a cellular model of memory in the mammalian hippocampus. While a number of behavioral tasks involving spatial learning have been useful in relating memory to the hippocampus, it has been difficult to directly relate hippocampal LTP to these memory functions.

In other mammalian systems, considerable progress has been made in achieving what seemed impossible in the late 1960s — identification of the circuit required for learning memory formation. Especially promising has been work on the circuits underlying two forms of Pavlovian classical conditioning in which simple stimuli, when associated with a noxious stimulus, acquire the capacity to elicit specific protective responses. One form of conditioning involves eyelid responses and depends on circuits involving the cerebellum, while another form involves so-called fear or defense responses and depends on circuits involving the amygdala. These are examples of systems that learn and store information implicitly, as discussed above. Another system in which some progress has been made in the mammalian brain involves learning about tastes that lead to illness. This also involves the amygdala, as well as certain regions of the cortex.

Studies of amygdala-dependent fear conditioning have been able to accomplish what studies of hippocampal dependent memory have had difficulty with — linking LTP to memory. By identifying a specific circuit involved in fear conditioning, and then studying both LTP and physiological changes that occur during learning in that circuit, it has been possible to obtain evidence that something like LTP occurs in amygdala regions during fear conditioning. Further, the site of plasticity has been pinpointed to a small set of neurons.

The molecular revolution in biology has spilled over into neuroscience and has provided many new tools for the pursuit of memory mechanisms. Particularly important has been the ability to alter the genetic composition of animals, especially flies and mice, allowing the manipulation of the function of individual genes and their proteins. This approach has led to the discovery that many of the same molecules involved in LTP in the hippocampus are also involved in the learning and memory of tasks that depend on the hippocampus. Furthermore, and even more remarkable, many of the molecules implicated in hippocampal plasticity are also implicated in plasticity in invertebrates. Interestingly, the tasks used to study associative learning in invertebrates is, like the tasks often used to study associative learning in vertebrates, classical conditioning and many of the same molecules have also been implicated in amygdala-dependent fear conditioning and amygdala LTP.

Some of the major trends of central importance to the study of learning and memory are as follows. Firstly, the tools for producing genetically altered animals are rapidly evolving, leading the ability to turn genes on and off at will, and even in select circuits. Secondly, the ability to screen genes and proteins that are activated during memory formation has emerged, but is still in an early stage of development. What is needed are improved ways to target this analysis to specific circuits and cells, and practical ways of analyzing the massive amounts of data that are generated. Thirdly, improved techniques for recording from multiple single neurons simultaneously are advancing our ability to understand how memory is encoded in neural activity. However, most work in memory physiology focuses on one brain region at a time. Given that each form of memory involves several regions, it will be interesting to use the new techniques to try to understand how memory is encoded across brain regions within a system. Furthermore, given that multiple systems encode memories during any given experience, it is important to begin to assess memory across systems, and to understand in more detail how such processes as emotion and attention alter memory storage within and across systems. Techniques for imaging the brain such as fMRI are also useful in this regard. These have mostly been applied to the human brain, but advances in nonhuman imaging should make possible interesting studies of both large scale (fMRI) and small scale (multiple single cell recording) in the same brain region. Advances in imaging techniques are also improving our ability to study the cell biology of the nervous system, especially to explore the molecules involved in synaptic function and plasticity. Finally, new studies are changing the way we think of that hallowed process — consolidation. This works suggests that not only is protein synthesis required during the consolidation of a memory but also each time the memory is retrieved. This is called reconsolidation, a term that raises as many questions as it answers.

It is becoming increasingly recognized that the molecular mechanisms of synaptic plasticity that are so pivotal to our understanding of learning and memory are also involved in many other key functions in the brain. For example, these same plastic mechanisms seem to be utilized during the formation and stabilization of synaptic connections during development. Following brain injury, for example as a consequence of stroke, there is a degree of recovery of brain function that may also involve similar plastic mechanisms. Various pathologies, such as epilepsy, involve plastic changes in the brain that are likely to usurp these mechanisms (e.g., kindling). At one extreme, aberrant activation of the plastic machinery may contribute to cell death during both acute and chronic conditions. Conversely, too little or inappropriate activation of plastic mechanisms may contribute to psychiatric conditions, such as schizophrenia and depression. The common link between many of these processes is the NMDA receptor, whose main physiological function is to initiate plastic changes at synapses. Therefore the study of the molecular basis of learning and memory impinges on many aspects of brain function in terms of health and disease.

Learning and memory are extremely active areas of research and, while much has been learned, there is still further work to do. The Learning and Memory Domain of *TheScientificWorldJOURNAL* will focus on synaptic plasticity relevant to all aspects of learning and memory, from behavioral and systems to cellular and molecular approaches. We will make every effort to ensure that this Domain is a rapid communication environment for papers that have been thoroughly peer reviewed. While not all papers can be published, we plan to be prompt, clear, and helpful in dealing with each submission. We have a distinguished team of Editors working on this Domain reflecting a range of research interests, and envisage that these will be reflected in the submissions that we receive. The Domain will work closely with other related Domains, including the Cognition and Higher Level Brain Function Domains, to achieve multidisciplinary and interdisciplinary exchange of information. We hope that the Learning and Memory Domain will become a premier forum for presenting the latest and most important findings in this exciting area of research.

**This article should be referenced as follows:**

LeDoux, J. and Collingridge, G. (2002) Founding editorial – Learning and Memory. *TheScientificWorldJOURNAL* **2**, 1685–1688.
